# A Maxim

**Published:** 1891-02

**Authors:** 


### A MAXIM.

### DEDICATED TO A DENTIST.

"Shoemaker, stick to your last!"
This venerable maxim comes out
Of the sage old days of the past,
In a word be it said,
Don't give up your trade
For one you know nothing about.

